# New Indicators for the Assessment and Prevention of Noise Nuisance

**DOI:** 10.3390/ijerph191912724

**Published:** 2022-10-05

**Authors:** Luca Fredianelli, Peter Lercher, Gaetano Licitra

**Affiliations:** 1 Institute of Chemical and Physical Processes of National Research Council, Via G. Moruzzi 1, 56124 Pisa, Italy; 2 Institute for Highway Engineering and Transport Planning of Graz University of Technology, Rechbauerstraße 12/II, 8010 Graz, Austria; 3 Environmental Protection Agency of Tuscany Region, Via Vittorio Veneto 27, 56127 Pisa, Italy

**Keywords:** noise indicators, noise metrics, psychoacoustic, nuisance, annoyance, sleep disturbance, peak noise, impulsive events, health related quality of life, health effects

## 1. Introduction

At present, health effects induced by prolonged noise exposure are widely studied to determine the most spread noise sources and their effects. Environmental epidemiology studies have mostly associated long-term exposure to high noise levels (>85 dB) in occupational (military, construction workers, and agriculture) and leisure settings with direct auditory effects, including hearing loss [1], tinnitus, and hyperacusis [2], whereas non-auditory effects are limited [3] due to occupational selection effects. On the other hand, long-term exposure to low–medium levels (45–65 dB) in general population settings is followed by a broad spectrum of non-auditory health effects [4,5]. Annoyance [6], sleep disturbance [7], cognitive impairment [8], behavioral and emotional disorders in children and adolescents [9], depression and anxiety in adults [10], stronger physiological stress reactions [11], and endocrine imbalance and cardiovascular disorders [12] are evidence-based effects. Overall, noise is among the leading risk factors for cardiovascular morbidity and mortality worldwide [13]. Usually, these studies regress the health effects of noise on acoustic exposure metrics, which is the average energetic dose over a long time period, such as equivalent continuous sound pressure level (L_eq_), with its A-weighted version L_Aeq_, or the day–evening–night level (L_den_).

Only recently, the scientific community has realized that health effect studies of long-term noise exposure should consider a broader spectrum of sound exposure features as well. Among those features are its intensity variation over time, the impulsivity of events, the frequency distribution, and psychoacoustics parameters. Peak levels, maximum levels, and variability can have a significative influence on nuisance perception, and citizens are known to complain more about single high levels rather than average exposure. This can be even more important for non-traffic-related sources such as leisure noise [2], or even less investigated sound sources such as ships [14] or wind turbines [15]. Generally, an incorrect metric may be the origin of flaws in dose–effect relationships for annoyance or sleep disturbance outcomes, which are mainly used for noise limit settings due to its importance at the population level.

This Special Issue was launched to promote a subject which is deserving of more attention: the study of new metrics, indicators or evaluation methods for noise exposure, and the relationship of noise with annoyance or other health effects, thus not relying only on an average noise exposure measure.

## 2. Summary of Published Papers

Most noise limits set by countries are expressed in absolute sound pressure levels, with only a few of them set with respect to the background noise level. This leads to the definition of sound emergence or, more generally, differential noise indicators. Many decades have passed since their introduction into legislation, when sound emergence was intended to limit the alterations of existing soundscapes which, in practice, raised several challenges both for the operators of noisy facilities, community, noise consultants, and authorities. Dutilleux et al. [16] established the concept of sound emergence as defined by international standardization in order to evaluate its relevance from different perspectives and to show that it was difficult to implement without bringing any benefit with respect to sound-pressure-level-based limit values on most occasions. The relevance of sound emergence was assessed from the point of view of perception and annoyance, in measurement practice and in development planning. Sound emergence seems to poorly predict audibility; the authors suggest considering the temporal and spectral characteristics of a specific source in the estimation of sound emergence, proposing source-specific choices of the metrics used for estimating total and residual sound pressure levels. Even if further research on the potential correlation between annoyance and sound emergence from specific sources is necessary, the best solution proposed is to combine an estimation of the audibility of specific sounds for the community and the estimation of a specific metric (Lspec).

Asensio et al. [17] investigated the scientific literature in research for the minimum set of indicators that would cover all physical dimensions of sound environments: energetic, spectral, and temporal dimensions, emergence, and source-related indicators. The extended set of indicators is intended to allow a more detailed analysis of the changes in noise environments related to confinement in the pandemic era and to a broader understanding the impact on sound environments of any policy achieved at the urban scale. In fact, noise indicators generally deal with sound environments considered as a whole, although they do not distinguish between the sound sources that compose it. Lockdowns imposed due to COVID-19 dramatically changed the noises emitted in terms of sound levels and by the different active sources. Reducing masking sounds made natural sounds perceptible again. Current indicators fail to represent this situation, although new source-orientated indicators would be able to quantify the presence of sources of interest. The authors conclude that the new physical indicators are associated with perceptual and health effects, but they will probably not be sufficient to represent the entire sound experience because sensitive and personal data or the connection between the sound environment and emotional evocations cannot be captured by physical indicators.

Differentiated studies for the singular type of sound sources were instead carried out by other authors published in this Special Issue, where most major sound sources have been investigated. From a point of view of the effects of noise on health, Petri et al. [18] evaluated the relationship of blood pressure and hypertension with noise produced by road, railway, airport, and leisure sources. In order to do so, noise measurements, blood pressure measurements, and a structured questionnaire were conducted on a significant set of patients. Noise exposure during the nights and diastolic blood pressure resulted correlated, with particular spikes for elders, moderately annoyed, noise-sensitive, without noise protection in the house and residents who usually did not close windows. In the investigated population, railway noise was the most impacting sound. The study also demonstrated an increase in the risk of hypertension in association with environmental noise.

Laboratory listening experiments were performed by Schäffer et al. [19], with the aim of searching for associations of annoyance and cognitive performance with the macro-temporal pattern of indoor exposure to noise emitted by road traffic. Relative quiet time and quiet time distribution were among the different metrics computed for the temporal pattern of the scenarios. Noise annoyance decreased with the increasing total duration of quiet periods, while shorter but regular breaks were less annoying than longer but irregular breaks. Different results were found for cognitive performance.

A lot of attention has been paid to interior noise in the present Special Issue. Li and Zheng [20] investigated passive noise control equipment as a means to control indoor environmental noise in contrast with the common applications of sound absorption materials and vibration isolation. According to the authors, active noise control has developed sufficiently to be effectively in avoiding the deficiencies of passive noise control, which are good mitigators for medium- and high-frequency noise but require a complex equipment and studies; generally, the low-frequency control effect is poor. In active noise control, loudspeakers are used to suppress noise in a specific area. Loudspeakers are omnidirectional; therefore, the sound pressure levels are reduced in the target area but are increased somewhere else. In their study, the authors calibrated a parametric array loudspeaker to achieve noise control in the target area in order to obtain a noise reduction topping 15.1 dB. The use of parametric array loudspeakers producing high-directivity sounds resulted to significantly reduce the noise interference to other adjacent areas while making the noise reduction areas more controllable.

Eventually, this solution would be beneficial even inside helicopter cabins, where Deacounu et al. [21] highlight the noise spectral components with measurements performed in different areas. High sound pressure levels were measured, with the urgency of reducing the exposure for passengers and crew. Main sources were identified in the transmission gear and the door area, with values during flight ranging from 97.2 dB(A) on the tail to 106.5 dB(A) under the transmission gear. Although the equivalent sound pressure levels vary spatially, the authors show that a much higher peak is registered at 2 kHz, and that this causes the most discomfort. This confirms once again how tonal sounds are more disturbing than others.

Railway noise and vibrations were investigated by Yan et al. [22], who performed measurements inside a metro train. Using A-weighted and linear sound pressure levels as metrics, and the FFT method for vibration measurements, the authors confirmed that interior sound levels increase sharply with the train’s speed, while acceleration levels of the floor and sidewall are not apparent. Moreover, floor vibration contributed to the low-frequency noise components of the interior noise, and the characteristics of the vehicle dominate the frequency peaks of the acceleration levels of the floor and sidewall.

Leisure noise, together with the other major sources, have been investigated innovatively by Peplow et al. [23], using big data, i.e., citizens’ tweets. The authors introduce a method, based on Python language, for estimating both the prevalence and location of noise annoyance. The open-access result produced would be also usable for the live monitoring of noise issues by means of tagging noise complaints.

Even port noise has been investigated in the Special Issue, with Schiavoni et al. [24] providing a review of recent findings in the scientific literature regarding port noise sources, an argument that has only quite recently been studied [25,26,27,28]. The database produced is expected to be useful to experts as inputs to the noise mapping phase, which is a fundamental step in the prevention of noise exposure. Recent decades have shown much focus from scientific community in this regard, with studies aimed at mitigating sound levels produced by almost all sources. Thus, quieter environments are expected. Unfortunately, Vukić et al. [29], by means of questionnaires on fishermen, showed that there are still sectors or environments where the impact of noise on health has not yet been accepted as a real danger and remains underestimated. Full social awareness can be reached only with proper education. In fact, the authors showed that when the test subjects underwent additional training, their practical knowledge and awareness of the noise impacts on health and society improved.

## 3. Conclusions

This Special Issue on the theme of the New Indicators for the Assessment and Prevention of Noise Nuisance has attracted the interest of authors from all over the world, publishing two reviews, two communications, as well as original research papers.

Progress has been made in the topic investigated, but it is still necessary to increase the awareness of the population, both in geographical terms and for workers in specific sectors, such as the marine industry.

It emerged that it is essential to carry out future studies that distinguish more between different sound sources with respect to their sound quality in terms of frequency, time pattern (fluctuation, emergence), and psychoacoustic indices, because a differential human reaction to sound sources is increasingly evident. More longitudinal studies are required. However, cross-sectional studies employing a more detailed soundscape description (including background) by competing sound indices are also useful to further the required knowledge to understand the human response in terms of the broad spectrum of potential adverse effects on health and quality of life.

## References

[1] Themann C.L., Masterson E.A. (2019). Occupational noise exposure: A review of its effects, epidemiology, and impact with recommendations for reducing its burden. J. Acoust. Soc. Am..

[2] Pienkowski M. (2021). Loud Music and Leisure Noise Is a Common Cause of Chronic Hearing Loss, Tinnitus and Hyperacusis. Int. J. Environ. Res. Public Health.

[3] Teixeira L.R., Pega F., Dzhambov A.M., Bortkiewicz A., Silva D.T.C., de Andrade C.A.F., Gadzicka E., Hadkhale K., Iavicoli S., Martínez-Silveira M.S. (2021). The effect of occupational exposure to noise on ischaemic heart disease, stroke and hypertension: A systematic review and meta-analysis from the WHO/ILO Joint Estimates of the Work-Related Burden of Disease and Injury. Environ. Int..

[4] Basner M., Babisch W., Davis A., Brink M., Clark C., Janssen S., Stansfeld S. (2014). Auditory and non-auditory effects of noise on health. Lancet.

[5] Persson-Waye K., van Kempen E.E.M.M. Non-auditory effects of noise: An overview of the state of the science of the 2017–2020 period. Proceedings of the International Commission on Biological Effects of Noise, ICBEN.

[6] Guski R., Schreckenberg D., Schuemer R. (2017). WHO environmental noise guidelines for the European region: A systematic review on environmental noise and annoyance. Int. J. Environ. Res. Public Health.

[7] Basner M., McGuire S. (2018). WHO environmental noise guidelines for the european region: A systematic review on environmental noise and effects on sleep. Int. J. Environ. Res. Public Health.

[8] Thompson R., Smith R.B., Bou Karim Y., Shen C., Drummond K., Teng C., Toledano M.B. (2022). Noise pollution and human cognition: An updated systematic review and meta-analysis of recent evidence. Environ. Int..

[9] Schubert M., Hegewald J., Freiberg A., Schubert M., Hegewald J., Freiberg A., Starke K.R., Augustin F., Riedel-Heller S.G., Zeeb H. (2019). Behavioral and Emotional Disorders and Transportation Noise among Children and Adolescents: A Systematic Review and Meta-Analysis. Int. J. Environ. Res. Public Health.

[10] Dzhambov A.M., Lercher P. (2019). Road traffic noise exposure and depression/anxiety: An updated systematic review and meta-analysis. Int. J. Environ. Res. Public Health.

[11] Daiber A., Kröller-Schön S., Frenis K., Oelze M., Kalinovic S., Vujacic-Mirski K., Kuntic M., Bayo Jimenez M.T., Helmstädter J., Steven S. (2019). Environmental noise induces the release of stress hormones and inflammatory signaling molecules leading to oxidative stress and vascular dysfunction-Signatures of the internal exposome. Biofactors.

[12] van Kempen E., Casas M., Pershagen G., Foraster M. (2018). WHO environmental noise guidelines for the European region: A systematic review on environmental noise and cardiovascular and metabolic effects: A summary. Int. J. Environ. Res. Public Health.

[13] Lim S.S., Vos T., Flaxman A.D., Danaei G., Shibuya K., Adair-Rohani H., Amann M., Anderson H.R., Andrews K.G., Aryee M. (2012). A Comparative Risk Assessment of Burden of Disease and Injury Attributable to 67 Risk Factors and 21 Risk Factor Clusters in 21 Regions, 1990–2010: A Systematic Analysis for the Global Burden of Disease Study 2010. Lancet.

[14] Badino A., Borelli D., Gaggero T., Rizzuto E., Schenone C. (2016). Airborne noise emissions from ships: Experimental characterization of the source and propagation over land. Appl. Acoust..

[15] Fredianelli L., Gallo P., Licitra G., Carpita S. (2017). Analytical assessment of wind turbine noise impact at receiver by means of residual noise determination without the wind farm shutdown. Noise Control. Eng. J..

[16] Dutilleux G., Gjestland T., Licitra G. (2019). Challenges of the use of sound emergence for setting legal noise limits. Int. J. Environ. Res. Public Health.

[17] Asensio C., Aumond P., Can A., Gascó L., Lercher P., Wunderli J.M., Licitra G. (2020). A taxonomy proposal for the assessment of the changes in soundscape resulting from the COVID-19 lockdown. Int. J. Environ. Res. Public Health.

[18] Petri D., Licitra G., Vigotti M.A., Fredianelli L. (2021). Effects of exposure to road, railway, airport and recreational noise on blood pressure and hypertension. Int. J. Environ. Res. Public Health.

[19] Schäffer B., Taghipour A., Wunderli J.M., Brink M., Bartha L., Schlittmeier S.J. (2022). Does the Macro-Temporal Pattern of Road Traffic Noise Affect Noise Annoyance and Cognitive Performance?. Int. J. Environ. Res. Public Health.

[20] Li Y., Zheng W. (2021). A Noise Control Method Using Adaptive Adjustable Parametric Array Loudspeaker to Eliminate Environmental Noise in Real Time. Int. J. Environ. Res. Public Health.

[21] Deaconu M., Cican G., Toma A.C., Drăgășanu L.I. (2021). Helicopter Inside Cabin Acoustic Evaluation: A Case Study—IAR PUMA 330. Int. J. Environ. Res. Public Health.

[22] Yan L., Chen Z., Zou Y., He X., Cai C., Yu K., Zhu X. (2020). Field study of the interior noise and vibration of a metro vehicle running on a viaduct: A case study in Guangzhou. Int. J. Environ. Res. Public Health.

[23] Peplow A., Thomas J., AlShehhi A. (2021). Noise annoyance in the UAE: A Twitter case study via a data-mining approach. Int. J. Environ. Res. Public Health.

[24] Schiavoni S., D’Alessandro F., Borelli D., Fredianelli L., Gaggero T., Schenone C., Baldinelli G. (2022). Airborne Sound Power Levels and Spectra of Noise Sources in Port Areas. Int. J. Environ. Res. Public Health.

[25] Fredianelli L., Bolognese M., Fidecaro F., Licitra G. (2021). Classification of noise sources for port area noise mapping. Environments.

[26] Borelli D., Gaggero T., Rizzuto E., Schenone C. (2016). Holistic control of ship noise emissions. Noise Mapp..

[27] Fredianelli L., Gaggero T., Bolognese M., Borelli D., Fidecaro F., Schenone C., Licitra G. (2022). Source characterization guidelines for noise mapping of port areas. Heliyon.

[28] Nastasi M., Fredianelli L., Bernardini M., Teti L., Fidecaro F., Licitra G. (2020). Parameters affecting noise emitted by ships moving in port areas. Sustainability.

[29] Vukić L., Mihanović V., Fredianelli L., Plazibat V. (2021). Seafarers’ Perception and attitudes towards noise emission on board ships. Int. J. Environ. Res. Public Health.

